# Good Health and Long Life

**Published:** 1915-06

**Authors:** 


### Good Health and Long Life.

The human body, like all other objective realities, is made up of matter. The ultimate constitution of matter is a subject which has engaged the deepest study and the most profound thought of many of the world's greatest thinkers for more than two thousand years, but until within recent years no general agreement has been reached concerning it. During the last few years, however, the general consensus of opinion among the world's greatest scientists or rather physicists, is, that in its ultimate constitution, matter is electricity. About four years ago Dr. W. D. Horne wrote as follows: "From considerations based (partly) on very elaborate mathematical calculations it is now maintained that matter is composed of electricity and nothing else. Electricity here is not considered a form of energy any more than water is a form of energy, but as a vehicle of energy, which can be moved from place to place, and whose energy must be in the form of motion or strain. In motion it constitutes current and magnetism; under strain it constitutes charge, and in vibration it constitutes light." (Science History of the Universe, Vol. 3, p. 18.)

We formerly regarded the atom as the smallest division of matter. While experimentation with colored solutions of a known concentration shows the hydrogen atom to be almost infinitesimal in size, it being about one-five hundredth millionth of an inch in diameter and weighing about one-ten billionth of a grain, yet our greatest physicists claim that the atom is made up of still smaller bodies known as electrons. "Sir Oliver Lodge describes the atom of matter as constituted of an individualized mass of positive electricity diffused uniformly over a space the size of an atom, perhaps spherical in shape. Throughout this small spherical space some eight hundred minute particles of negative electricity, all exactly alike, are supposed to be scattered, flying vigorously about, each repelling every other and yet all contained within their orbits by the mass of positive electricity. The positive electricity is very much attenuated and constitutes perhaps only about 1 per cent of the mass of the atom, while the negative electrons are correspondingly dense and so inconceivably small that the eight hundred are less crowded in their atom than are the planets in the solar system. Atoms of different kinds of matter are supposed to be constructed in the same general manner and of the same kind of electrons, but the number of electrons in an atom is proportional to the atomic weight of the element. Thus oxygen would have sixteen times as many electrons in its atom as has hydrogen. When the crowding becomes excessive, as in the very heavy atoms of uranium (the heaviest substance known), thorium and radium having atomic weights well over two hundred, the atoms become radio-active, probably due to numerous collisions between the electrons, some of which are being constantly shot away." (Vide supra, p. 19.) Except in some of the newer gases the atoms never exist alone but combine in pairs or groups to form molecules.—*Charlotte Medical Journal.*

